# Effects of copper on leaf membrane structure and root activity of maize seedling

**DOI:** 10.1186/s40529-014-0047-5

**Published:** 2014-05-27

**Authors:** Jiao Jiao Liu, Zhen Wei, Jia Hui Li

**Affiliations:** 1 School of Life Sciences of East China Normal University, No.500, Dongchuan Rd, Shanghai, 200241 China; 2 No.2 Secondary School Attached to East China Normal University, No.555, Chenhui Rd, Shanghai, 201203 China

**Keywords:** Copper, Maize, Membrane structure, Root activity

## Abstract

**Background:**

Copper is an important heavy metal pollutant, with strong toxicity and great harm, which is easy to accumulate in the plant body and is difficult for degradation. This paper adopts medium culture method, taking “Zheng Dan 958” maize seedlings as sample materials. With different copper ion concentration gradients for the simulation of metal copper stress on maize seedlings, it explored the effects on the membrane structure (POD activity, MDA content, membrane permeability) and root activity.

**Results:**

POD activity increases dramatically when the copper concentration is over 10 μmol/L. MDA content increases sharply when the copper concentration is over 1000 μmol/L, showing a rising trend. Membrane permeability increases greatly when the copper concentration is over 100 μmol/L. Root activity decreases significantly when the copper concentration is 100 μmol/L, showing a clear downward trend.

**Conclusions:**

The copper concentration of 1000 μmol/L has exceeded the maize seedling tolerance to copper, and the activities of protective enzymes of maize seedlings are inhibited. Cell membrane lipid peroxidation has caused serious damage on the structure and function of membrane. Structure of root cells of maize seedling is also damaged, reducing the root activity, so the maize is irreversible hurt.

**Electronic supplementary material:**

The online version of this article (doi:10.1186/s40529-014-0047-5) contains supplementary material, which is available to authorized users.

## Background

Heavy metals are main pollutants, which have potential harm on the environment. Along with the development of industry, heavy metal pollution has become a serious problem. Copper mining, discharge of the three wastes of smelting plant, agricultural chemicals containing copper and the discharge of domestic sewage, the soil copper content is several times of the original content in soil (Besnard et al. [2001]; Brun et al. [2001]). Copper pollution has become a worldwide problem in recent years: copper is not degraded by microorganisms in the soil, so it is generally difficult to eliminate the pollution; the high rate of copper residue in the soil can cause many serious problems to the environment and human health; copper is an important heavy metal pollutant, with strong toxicity and great harm, which is easy to accumulate in the plant body and is difficult for degradation. Copper pollution problem has aroused extensive attention.

However, copper has played an important role on plant physiological metabolism: as one of the 9 kinds of trace elements in plants which are necessary to normal life activities, copper is a part of oxidoreductase (such as polyphenol oxidase, cytochrome oxidase, ascorbic acid oxidase), the lack of copper will directly affect the metabolism of plant in the process of growth and development. But copper has a cumulative nature (Chang et al. [2000]); plants’ absorption of copper ions will be fixed in the root cortex, affecting the absorption of other nutrients. Excessive copper will produce toxic effects to plants, facilitating the formation of reactive oxygen species, causing cell oxidative damage and increasing membrane permeability (Kong et al. [1999]; Zhang et al. [1997, 2006]; Reboredo and Henriques [1991]; Eleftheriou and Karataglis [1989]; Madejonb et al. [2009]), which will change plant antioxidative enzyme system (Scandalous [1993]).

Maize (*Zea mays L.*) belongs to Maydeae group of Poaceae family, which is widely distributed in China. It is one of the main foods of people in North China, Southwest Mountain and other dry Gulch area. Many scholars have studied the physiological and ecological effects of different heavy metals on maize. Kong et al. ([1999]) studied on the change of permeability and protective enzyme activity of maize seedlings cell membrane under Cd^2+^ stress (Kong et al. [1999]). The results showed that with the increase of cadmium concentration, POD activity, MDA content and the membrane permeability increased, the growth of the seedlings was inhibited. Zhang et al. ([1997]) conducted the hydroponics experiment to study the effects of Hg^2+^ on the oxidative defense system of maize seedlings lipid (Zhang et al. [2006]). Results showed that: after Hg^2+^ treatment, maize leaves membrane permeability and MDA content increased significantly; the activity of POD significantly decreased. By nutrient solution culture experiment, Zhang et al. ([2006]) studied root vigor and activity of antioxidant enzymes of different varieties of maize seedling stressed by Pb^2+^ (Zhang et al. [1997]). Results showed that: under the impact of Pb^2+^, root vigor decreased. As Pb^2+^ concentration increasing, POD activity was increased and then decreased. However, the stress effect of heavy metal copper on maize membrane structure and root activity has not been reported.

This paper adopts medium culture method, taking “Zheng Dan 958” maize seedlings as sample materials. With different copper ion concentration gradients (0, 1, 10, 100, 1000, 5000, 10000 μmol/L) for the simulation of metal copper stress on maize seedlings, it explored the effects on the membrane structure (POD activity, MDA content, membrane permeability) and root activity. It reveals the effect of copper stress in maize, which will provide certain theory reference for the agricultural production and rational application of trace element copper.

## Methods

### Materials

Tested material is “Zheng Dan 958” maize. Select plump and uniform maize seeds, after 0.2% K_2_MnO_4_ immersion disinfection of 20 min, wash with clean water for several times, put into the digital electric incubator in distilled water for 8 h. The temperature of the incubator is set at 30°C. Soaked seeds are planted in pots containing an equal amount of vermiculite, which is wet with distilled water. Keep the fixed seed spacing and embryo direction when planting, with 30 seeds in one basin and a total of 28 basins. Put a layer of dry vermiculite in each basin. After planting, put basins into light incubator (without illumination, temperature of 25°C) in culture. Turn on the light after 2d in the 4 level of light intensity (2 time intervals a day, total 14 h under illumination) and timely pour distilled water. After 7d, select 15 seedlings of uniform growth. Keep culturing in light incubator and conduct copper stress processing after 4d.

Use CuSO_4_·5H_2_O (A. R.) to make Hoagland culture medium of different copper concentrations: 0, 1, 10, 100, 1000, 5000, 10000 μmol/L, pour equivalent Hoagland medium of different copper concentrations on plants (each treatment has 4 replicates) and label them, culture for 12d and pour medium for 3 times. 12d later, measure each physiological index.

### Test methods

#### Peroxidase (POD) activity determination

According to the literature (Zhang et al. [2000]), take 0.2 g tested plant materials leaves treated with different concentrations of copper, chop into a mortar, add a little quartz sand and solution of 2 mL 20 mmol/L KH_2_PO_4_ into the mortar; grind into homogenate, put it into a centrifuge tube. Then add 3 mL 20 mmol/L KH_2_PO_4_ solutions to rinse the mortar, put the solution into a centrifuge tube (4000 r/min centrifugal for 15 min). Supernatant is collected and preserved in the cold. Extract the residues with 5 mL KH_2_PO_4_ solution for another time, merge the two supernatant. The 3 mL reaction mixture (50 mL 100 mmol/L pH = 6.0 phosphate buffer; 19 μL 30% H_2_O_2_; 28 μL guaiacol) and 1 mL enzyme are add in the cuvette for the determination of enzyme activity. Meanwhile, 3 mL reaction mixed solution and 1 mL KH_2_PO_4_ solution is the control group. Immediately turn on the stopwatch, measure OD at the spectrophotometer 470 nm wavelength; read OD value every minute. OD value changes indicate that the enzyme activity, which is represented by ΔA_470_/[min · FW (g)].

#### Malondialdehyde (MDA) content determination

Take 0.3 g tested plant materials leaves treated with different concentrations of copper, chop into a mortar, add 2 mL 10% TCA and a small amount of quartz sand, grind into homogenate and pour into the centrifugal tube, add 6 mL TCA to rinse mortar, put the solution into a centrifuge tube (4000 r/min centrifugal for 10 min), supernatant is extracted as sample. Take 2 mL extract, add into 2 mL 0.6% TBA solution, mix and plug in the tube, seal with plastic wrap, place in boiling water for 30 min and then cool it rapidly. The supernatant is taken for the determination of OD value at 532 nm and 450 nm. The control group is the TCA solution. According to the formula C(μmol/L) = 6_·_45 × *A*_532_ – 0_·_56 × *A*_450_, calculate the MDA concentration, then calculate the content of MDA per gram (μmol/g).

#### Membrane permeability determination

Take 1.0 g tested plant leaf treated by different concentrations, put into a small triangle flask, add 20 mL distilled water, seal with plastic wrap, shake so that leaves are all immersed in distilled water, stand for 3 h at room temperature, measure the electrical conductance value (*S*_1_). Then it is placed in boiling water for 20 min, cool and measure the conductance value (*S*_2_), measure the electrical conductance value of distilled water (*S*_3_). Use $\frac{S_{1}-S_{3}}{S_{2}-S_{3}}\;\times \;100\%$ to express the relative membrane permeability (%).

#### Root activity determination

Prepare 0, 0.005%, 0.01%, 0.02%, 0.03%, 0.04% TTC solution, take 5 mL of each into graduated test tubes, take 5 mL ethyl acetate and a small amount of Na_2_S_2_O_4_ (approximately 2 mg, same quantity in each tube), shake sufficiently to produce red TTF, transfer to the ethyl acetate layer; after the colored liquid separation, add 5 mL of ethyl acetate, shake and rest, take the upper ethyl acetate solution, with a blank as the reference, to determine OD of the solution in the spectrophotometer 485 nm, then with TTC concentration as the abscissa, OD as ordinate, draw standard curve. TTC standard curve is *y* = 0.001*x* + 0.0034.

Take 0.5g plant root samples treated by different concentrations of copper; immerse in 10 mL beaker with the mixed liquid of 0.4% TTC and 66 mmol/L phosphate buffer solution (pH = 7.0), keep at 37°C for 3 h, then add 1 mol/L of sulfuric acid 2 mL to terminate reaction. Remove the root, carefully wipe it and grind with 2 mL ethyl acetate and a small amount of quartz sand in a mortar to extract TTF, use a small amount of ethyl acetate to wash the residue for 2 ~ 3 times, then pour into the test tube, finally add the ethyl acetate to scale, use a spectrophotometer for 485 nm colorimetry, taking the blank test (with sulfuric acid, then add the root samples) as reference, readout the OD. According to the standard curve of *y* = 0.001*x* + 0.0034, the reducing amount of TTC can be obtained. According to the reduction amount of TTC, we can get the reducing strength of TTC.

1 $$\mathrm{Reducing}\;\mathrm{Strength}\;\mathrm{of}\;\mathrm{TTC}\;=\;\frac{\mathrm{Reduction}\;\mathrm{Amount}\;\mathrm{of}\;\mathrm{TTC}\left(\mathrm{mg}\right)}{\mathrm{Weight}\;\mathrm{of}\;\mathrm{Root}\;\mathrm{Sample}\left(g\right)\times \mathrm{Time}\left(h\right)}$$

## Results and discussion

### Influence of peroxidase (POD) activity of copper stress on maize seedling leaf

**Figure 1:**
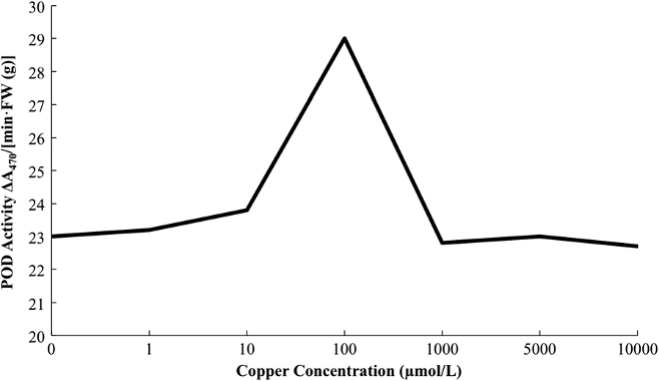
**Influence of POD activity of copper stress on maize seedling leaf.**

A lot of researches show that the essence of damage of membrane structure is a process of membrane lipid peroxidation. Lipid peroxidation is the harmful reaction of biological free radical (mainly active oxygen) on unsaturated fatty acid and some protective enzyme system can clean harmful reactive oxygen species (ROS) (Chen [1989, 1991]). POD is an important protective enzyme of plant defense against membrane lipid peroxidation, which is an important enzyme to clean H_2_O_2_ and many organic hydroperoxides. In the organization with little number of catalase content or low H_2_O_2_ content, it can replace the catalase peroxidase to eliminate H_2_O_2_ (Reddy et al. [2005]) and its activity can reflect the toxic symptoms of plant. Figure 1 shows that: with the deepening degree of copper stress, POD activity increases slowly. Under the copper concentrations of 1 μmol/L and 10 μmol/L, the gap of POD activity and control group is not big, which increases by 2% and 4.5%. The toxic level of maize seedlings is not high. The body produces reactive little oxygen. But when the copper concentration is over 10 μmol/L, POD activity increases dramatically. When the copper concentration is 100 μmol/L, POD activity is the biggest, increases by 26.8% than the control. When the maize seedlings are under the high concentrations of copper stress, the injured degree aggravates, the body produces more active oxygen and the seedlings make positive protection. The antioxidant capacity of maize seedlings increases because of adaption. H_2_O_2_ is deposed into H_2_O to resist the poison.

### Influence of malondialdehyde (MDA) content of copper stress on maize seedling leaf

**Figure 2:**
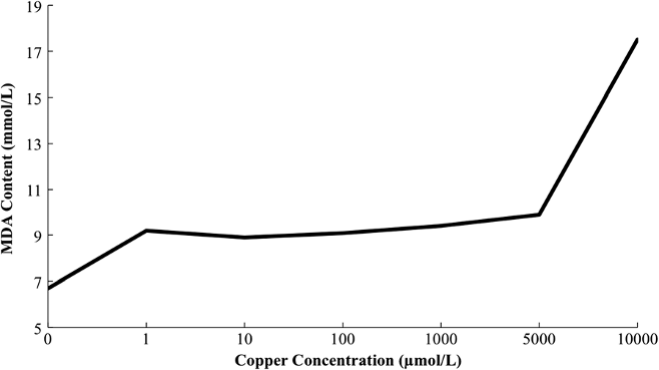
**Influence of MDA content of copper stress on maize seedling leaf.**

Copper stress results in large numbers of active oxygen free radicals in leaves of maize seedlings, causing cell membrane lipid peroxidation, damaging the normal structure and function of the membrane. MDA is one of the products of cell membrane lipid peroxidation, the combination of MDA and protein can cause protein intramolecular and intermolecular cross-link, which will seriously damage the biomembrane. Its content can react the level of cell membrane lipid peroxidation and the damage degree of cell membrane reaction. MDA content changes are often taken as the parameter of lipid peroxidation of cell membrane under adversity. Figure 2 shows: with the deepening degree of copper stress, MDA content first increases gradually before the trend. When the copper concentration reaches 1000 μmol/L, MDA content has little difference compared with control group, when the copper concentrations are 1, 10, 100, 1000 μmol/L, MDA content increases by 15.5%, 10.7%, 12.8% and 12.6%. That shows the low level of damage of cell membrane lipid peroxidation on cell membrane. When the copper concentration is 1000 μmol/L, MDA content increases sharply. When the copper concentrations are at 5000 μmol/L and 10000 μmol/L, MDA content increases by 42.6% and 144.4%, respectively. That means the high concentrations of copper stress can cause significant damage to the structure and function of cell membrane. Leaves produce oxidative stress, lipid peroxidation of cell membrane increases, MDA and other harmful products of membrane lipid peroxidation are produced, which may be an important mechanism of copper stress on plant growth.

### Influence of membrane permeability of copper stress on maize seedling leaf

Cell membrane is selectively permeable membrane, which can control and adjust the transport and exchange of intracellular substances. At the cellular level, it is the first site of stress injury. The permeability values reflect the amount of soluble substance leakage within the cell membrane, so is the evaluation index of the reaction of plants to environmental damage. Copper stress’ most direct harm to the plant cell is the structure and function of cell membrane, with the membrane permeability increases, membrane stability decreases, passive leakage of ion cells and macromolecules. Therefore, the increased membrane penetrability is the direct evidence of cell membrane damage.

**Figure 3:**
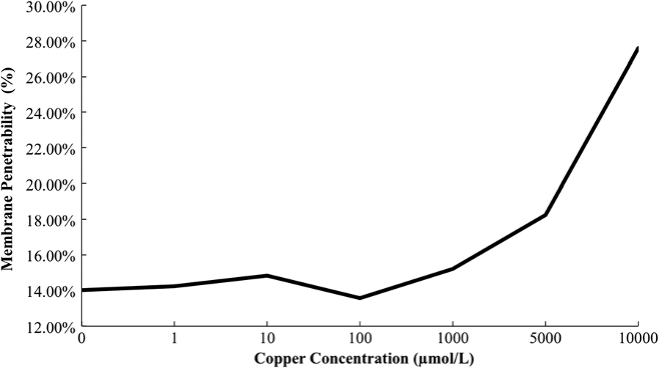
**Influence of membrane permeability of copper stress on maize seedling.**

Figure 3 shows that: with the deepening degree of copper stress, electrolyte leakage rate of maize seedlings increases slowly. Before the copper concentration reaches 100 μmol/L, membrane permeability changes little compared with control group. When the copper concentrations are 1, 10, and 100 μmol/L, membrane permeability increases by 1.9%, 4.1% and 1.3%, respectively. The structure and function of cell membrane have not yet been damaged. But when the copper concentration is over 100 μmol/L, membrane permeability increases rapidly, which shows a rising trend. When copper concentrations are 1000 μmol/L, 5000 μmol/L and 10000 μmol/L, membrane permeability increases by 19.5%, 47.1% and 104.8%, respectively. That means the high concentrations of copper stress can damage the structure and function of cell membrane in leaves of maize seedlings severely, promoting the electrolyte leakage.

Figure 3 also shows that although the change pattern of leaf MDA content and membrane permeability increases slowly at first and then increases sharply, the increasing amplitude is different. For membrane permeability, when the copper concentration is 1000 μmol/L, it increases significantly by 19.5% than that of the control group. For MDA content, when the copper concentration is 5000 μmol/L, it increases significantly by 42.6%. That means there are many factors causing the damage of cell membrane. Cell membrane lipid peroxidation is just one aspect. Certainly there are other factors leading to the increase of membrane permeability. Maybe copper, destroying the structure and function of membrane, due to membrane protein inactivation and degeneration causes it.

### Influence of root activity of copper stress on maize seedling leaf

Heavy metal pollutants can directly affect plant roots, while root activity can reflect the quality and metabolic status of root development in a certain extent, so the activity of root can be used to measure the degree of injury of root.

**Figure 4:**
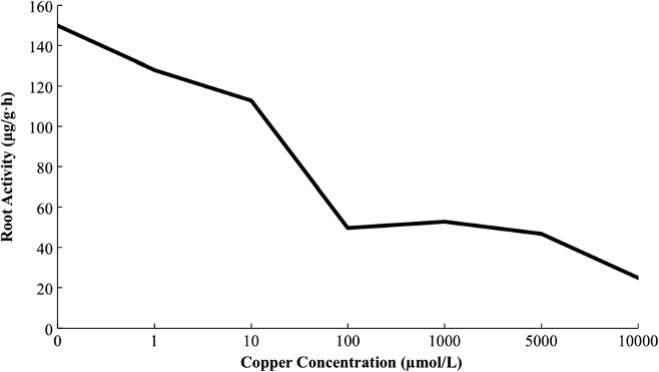
**Influence of root activity of copper stress on maize seedling leaf.**

Figure 4 shows that: copper stress damage on the root of maize seedling appears earlier. It is visible that root is the worst part of heavy metal stress. When the copper concentration is 1 μmol/L, the root activity begins to decrease by 18.3%. When the copper concentration is 10 μmol/L, it reduces to the maximum level, decreases by 62.7% comparing to the control group, which means that maize seedling roots have suffered serious damage. When copper concentration is 100 μmol/L, with increasing concentration, root activity decreases slowly.

## Conclusion

Copper is one of essential trace element for normal growth and development of plants, which takes part in various biochemical and physiological process in plant (Sudo et al. [2008]; Burkhead et al. [2009]). However, excessive copper can be toxic to plant cells, or even lead to cell death. An important feature of copper toxicity to plants is to produce oxidative stress, leading to the formation of harmful ROS. Under normal conditions, plants can effectively clean out ROS, protecting cells from damage. But in the face of adversity condition, when ROS is produced faster than the ability of plants to scavenge active oxygen, it will cause the damage (Li and Mei [1989]; Elastner [1982]; Chris et al. [1992]). POD can clear harmful reactive oxygen species, so as to protect the membrane system. Under the condition of this experiment, with the increasing of copper concentration, POD activity increases slowly. Before the copper concentration reaches 10 μmol/L, POD activity changes little relative to control group. But when the copper concentration is over 10 μmol/L, POD activity increases dramatically; when the copper concentration is up to 100 μmol/L, POD activity is the highest. Increased peroxidase activity is the stress response of plants, indicating that 100 μmol/L is the harmful concentrations on maize growth.

The plant cell membrane system is the interface and barrier for exchange of substance and information exchange of plant and environment, which plays an important role in maintaining cell microenvironments and normal metabolism. Its stability is the basic of normal physiological function. The copper stress can induce cell membrane lipid peroxidation and membrane structure and functional damage. Malondialdehyde (MDA) is one of the products of the cell membrane lipid peroxidation. Its content can reflect the degree of cell membrane lipid and membrane oxidative damage. It is found in the present study, when the copper concentration is over 1000 μmol/L, MDA content increases sharply, which shows a rising trend.

Copper toxicity-induced ROS directly or indirectly can attack the protein, lipid and other biological macromolecules, causing oxidation or degradation, leading to the increase of biological membrane permeability (Sharma and Dietz [2009]) and the stress intensity is related to the degree of increase of membrane permeability. Under the experimental condition, when the copper concentration is over 100 μmol/L, membrane permeability increases sharply. Under copper concentration ≥100 μmol/L, cell plasma membrane damage of maize seedling leaf is very serious.

Maize root is exposed to the damage of heavy metal firstly in the whole plant. It is also under the worst influence of copper stress. After root cell membrane being hurt, intracellular ion and organic substance leak greatly. External Cu^2+^ would take the opportunity to enter the cell, resulting in physiological metabolism disorder (Jiang and Zhao [2001]). It can be seen from the results of this study: Copper Stress has obvious effects on physiological and biochemical characteristics of maize seedling roots. When the copper concentration is 100 μmol/L, maize root activity decreases significantly, showing a clear downward trend. In addition, we can also infer that with deeper damage to maize roots, its absorptive capacity will continue to decrease, which will lead to plant aboveground nutrient deficiency and further accelerate the physiological metabolism disorder.

According to the variation curve of POD activity, MDA content, membrane permeability and root activity of maize seedlings under copper stress, the copper concentration of 1000 μmol/L is beyond the tolerance limit of maize seedlings. The activity of protective enzymes of maize seedlings is inhibited. Cell membrane is under serious lipid peroxidation, which damages the structure and function of membrane. Structure of root cells of maize seedling is damaged, reducing the root activity. Maize seedlings are under irreversible damage.

## Authors’ original submitted files for images

Below are the links to the authors’ original submitted files for images. Authors’ original file for figure 1 Authors’ original file for figure 2 Authors’ original file for figure 3 Authors’ original file for figure 4
