# Supplementary figures and images for: Effects of copper on leaf membrane structure and root activity of maize seedling
Source: Bot Stud. 2014 May 27;55:47. doi: 10.1186/s40529-014-0047-5 (PMC5432969; doi:10.1186/s40529-014-0047-5)

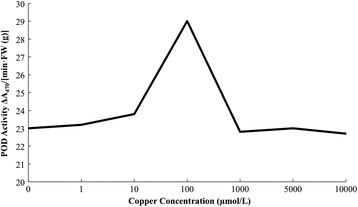

Supplement: Supplementary file 1 — Authors’ original file for figure 1 [file 40529_2014_47_MOESM1_ESM.gif]

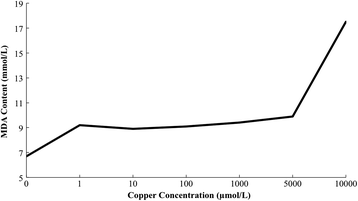

Supplement: Supplementary file 2 — Authors’ original file for figure 2 [file 40529_2014_47_MOESM2_ESM.gif]

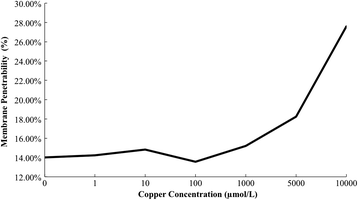

Supplement: Supplementary file 3 — Authors’ original file for figure 3 [file 40529_2014_47_MOESM3_ESM.gif]

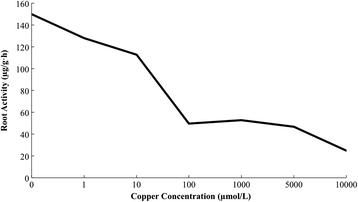

Supplement: Supplementary file 4 — Authors’ original file for figure 4 [file 40529_2014_47_MOESM4_ESM.gif]
